# The Effects of Acute Hydrogen Sulfide Poisoning on Cytochrome P450 Isoforms Activity in Rats

**DOI:** 10.1155/2014/209393

**Published:** 2014-03-26

**Authors:** Xianqin Wang, Mengchun Chen, Xinxin Chen, Jianshe Ma, Congcong Wen, Jianchun Pan, Lufeng Hu, Guanyang Lin

**Affiliations:** ^1^Analytical and Testing Center, Wenzhou Medical University, Wenzhou 325035, China; ^2^The First Affiliated Hospital, Wenzhou Medical University, Wenzhou 325000, China

## Abstract

Hydrogen sulfide (H_2_S) is the second leading cause of toxin related death (after carbon monoxide) in the workplace. H_2_S is absorbed by the upper respiratory tract mucosa, and it causes histotoxic hypoxemia and respiratory depression. Cocktail method was used to evaluate the influences of acute H_2_S poisoning on the activities of cytochrome P450 isoforms CYP2B6, CYP2D6, CYP3A4, CYP1A2, CYP2C19, and CYP2C9, which were reflected by the changes of pharmacokinetic parameters of six specific probe drugs, bupropion, metoprolol, midazolam, phenacetin, omeprazole, and tolbutamide, respectively. The experimental rats were randomly divided into two groups, control group and acute H_2_S poisoning group (inhaling 300 ppm for 2 h). The mixture of six probes was given to rats by oral administration and the blood samples were obtained at a series of time points through the caudal vein. The concentrations of probe drugs in rat plasma were measured by LC-MS. The results for acute H_2_S poisoning and control groups were as follows: there was a statistically significant difference in the AUC and *C*_max_ for bupropion, metoprolol, phenacetin, and tolbutamide, while there was no statistical pharmacokinetic difference for midazolam and omeprazole. Acute H_2_S poisoning could inhibit the activity of CYP2B6, CYP2D6, CYP1A2, and CYP2C9 in rats.

## 1. Introduction

Hydrogen sulfide (H_2_S) is one of the major toxic gases [1]; it is the second leading cause of toxin related death (after carbon monoxide) in the workplace [2]. H_2_S has a specific gravity of 1.19 and the characteristic odor of rotten eggs [3]. H_2_S is poisonous, and accidents may occur on exposure to natural gas, volcanic gas, and industrial waste [4]. Accidents have been reported in chemical processing plants [5, 6] and sewage disposal facilities [7–10] and with the ingestion of sulfur products [11, 12]. Acute toxicity of H_2_S involves mainly the central nervous system and lungs [13]. It may cause variable neurologic symptoms such as dizziness, headache, poor coordination, and brief loss of consciousness after exposure to high concentrations of H_2_S. If exposure is transient, recovery is usually complete and rapid. However, in some instances, prolonged or severe exposure leads to a fatal outcome or permanent sequelae [14, 15].

Cytochrome P450s are thiolate-ligated heme enzymes that use dioxygen and the formal equivalents of molecular hydrogen (2H+ and 2e−) to functionalize a wide range of biologically active compounds [16, 17]. Cytochrome P450 (CYP) is the most important drug metabolizing enzyme family contributing to the metabolism of the majority of drugs in humans [18–20]. CYP450 enzymes reduce or alter the pharmacodynamic activity of many drugs and are involved in ~80% of oxidative drug metabolism and 50% of elimination of commonly used drugs [21]. The main isoenzymes are CYP1A2, 2C9, 2D6, and 3A4; and ~20–25% of the population, depending on ethnic background, has genetic differences in drug metabolism by CYP450 [22]. In order to assess various individual CYP450 activities, probe drugs have been widely used in many clinical investigations in the field of drug metabolism and pharmacogenetics [23–25]. Probe drug is one kind of compound specially catalyzed by CYP isoforms, and the activities of CYP isoforms can be reflected by the metabolic rate of probe drug. As several CYP isoforms are involved in drug metabolism, the cocktail approach was developed.

At present, the study of H_2_S toxicology mainly focuses on the central nervous system and cardiovascular system [26–32]. To our knowledge, there are few reports about the hepatic toxicity of H_2_S. In this paper, cocktail probe drugs approach is used to evaluate the induction or inhibition effects of H_2_S on the activities of rats CYP450 isoforms such as CYP2B6, CYP2D6, CYP3A4, CYP1A2, CYP2C19, and CYP2C9, which are reflected by the changes of pharmacokinetic parameters from 6 specific probe drugs, bupropion, metoprolol, midazolam, phenacetin, omeprazole, and tolbutamide, and then to provide a guidance for rational clinical oral administration after acute H_2_S poisoning.

## 2. Material and Methods

### 2.1. Chemicals and Reagents

Bupropion, metoprolol, midazolam, phenacetin, omeprazole, tolbutamide (all >98%), and the internal standard carbamazepine (IS) were purchased from Sigma-Aldrich Company (St. Louis, USA). HPLC grade acetonitrile and methanol were from Merck Company (Darmstadt, Germany). All other chemicals were of analytical grade. Ultrapure water (resistance > 18 mΩ) was prepared by a Millipore Milli-Q purification system (Bedford, USA).

### 2.2. Animals

Male Sprague-Dawley rats (250 ± 20 g) were obtained from Shanghai SLAC Laboratory Animal Co., Ltd. The animal license number was SCXK (Shanghai) 2012-0005. All twenty rats were housed at Wenzhou Medical University Laboratory Animal Research Center. Animals were housed under controlled conditions (22°C) with a natural light-dark cycle. All experimental procedures were conducted according to the Institutional Animal Care guidelines and approved ethically by the Administration Committee of Experimental Animals, Laboratory Animal Center of Wenzhou Medical University.

### 2.3. Instrumentation and Conditions

Bruker Esquire HCT mass spectrometer (Bruker Technologies, Bremen, Germany) equipped with a 1200 Series liquid chromatograph (Agilent Technologies, Waldbronn, Germany) and controlled by ChemStation software (Version B.01.03 [204], Agilent Technologies, Waldbronn, Germany) was used.

Chromatographic separation was achieved on a 150 mm × 2.1 mm, 5 *μ*m particle, Agilent Zorbax SB-C18 column at 30°C. A gradient elution programme was conducted for chromatographic separation with mobile phase A (0.1% formic acid in water) and mobile phase B (acetonitrile) as follows: 0–4.0 min (10-80% B), 4.0–8.0 min (80-80% B), 8.0–9.0 min (80-10% B), and 9.0–13.0 min (10-10% B). The flow rate was 0.4 mL/min.

The quantification was performed by the peak area method. The determination of target ions was performed in selective ion monitoring mode (*m*/*z* 240 for bupropion, *m*/*z* 268 for metoprolol, *m*/*z* 326 for midazolam, *m*/*z* 180 for phenacetin, *m*/*z* 198 for omeprazole, *m*/*z* 271 for tolbutamide, and *m*/*z* 237 for IS) and positive ion electrospray ionization interface. Drying gas flow was set to 7 L/min and temperature to 350°C. Nebuliser pressure and capillary voltage of the system were adjusted to 25 psi and 3,500V, respectively.

### 2.4. Preparation of Standard Solutions

Stock solutions of 1.0 mg/mL for each of bupropion, metoprolol, midazolam, phenacetin, omeprazole, tolbutamide, and IS were prepared in methanol. The working standard solutions of each analyte were prepared by serial dilution of the stock solution with methanol. All of the solutions were stored at 4°C and brought to room temperature before use.

The calibration standards were prepared by spiking blank rat plasma with appropriate amounts of bupropion, metoprolol, midazolam, phenacetin, omeprazole, and tolbutamide. Calibration plots of each probe drug were constructed in the range 10–2000 ng/mL for plasma (10, 20, 50, 100, 200, 500, 1000, and 2000 ng/mL). Quality-control (QC) samples were prepared by the same way as the calibration standards, with three different plasma concentrations (20, 200, and 1600 ng/mL). The analytical standards and QC samples were stored at −20°C.

### 2.5. Pharmacokinetic Study

Twenty male Sprague-Dawley rats (250 ± 20 g) were randomly divided to control group and acute H_2_S poisoning group (*n* = 8); the rats were placed in triad infected ark which was with the H_2_S detector and passed into a certain concentration of H_2_S gases to create a model of acute H_2_S poisoning. The acute H_2_S poisoning group rats were exposed to 300 ppm H_2_S for 2 h. Control animals were maintained under similar conditions, but without the H_2_S exposure. Rats were allowed to eat and drink ad libitum except during the 2 h exposure.

After completing the modeling, the acute H_2_S poisoning and control rats were given the mixed six probe drugs with oral administration. The administration dose of the probe drugs bupropion, metoprolol, midazolam, phenacetin, omeprazole, and tolbutamide were 10 mg/kg, 10 mg/kg, 10 mg/kg, 10 mg/kg, 10 mg/kg, and 1 mg/kg, respectively.

Blood samples (0.3 mL) were collected from the tail vein into heparinized 1.5 mL polythene tubes at 5, 15, and 30 min and 1, 1.5, 2, 3, 4, 5, 6, 8, 12, 24, and 48 h after oral administration of probe drugs. The samples were immediately centrifuged at 8000 r/min for 5 min, and 100 *μ*L plasma was obtained for each sample.

The plasma samples were extracted and measured by LC-MS. In a 1.5 mL centrifuge tube, an aliquot of 10 *μ*L of the internal standard working solution (2.0 *μ*g/mL) was added to 0.1 mL of collected plasma sample followed by the addition of 0.2 mL of acetonitrile-methanol (v/v, 9 : 1). After the tube was vortex-mixed for 1.0 min, the sample was centrifuged at 15000 rmp for 10 min. The supernatant (2 *μ*L) was injected into the LC-MS system for analysis.

Plasma probe drugs concentration versus time data for each rat was analyzed by DAS software (Version 3.0, Drug Clinical Research Center, Shanghai University of TCM, and Shanghai BioGuider Medicinal Technology Co., Ltd., China). The pharmacokinetic parameters of the test group and control group probe drugs with the *t*-test inspection were analyzed by SPSS l8.0 statistical software. A *P* < 0.05 was considered as statistically significant.

## 3. Results

### 3.1. Method Validation

**Figure 1:**
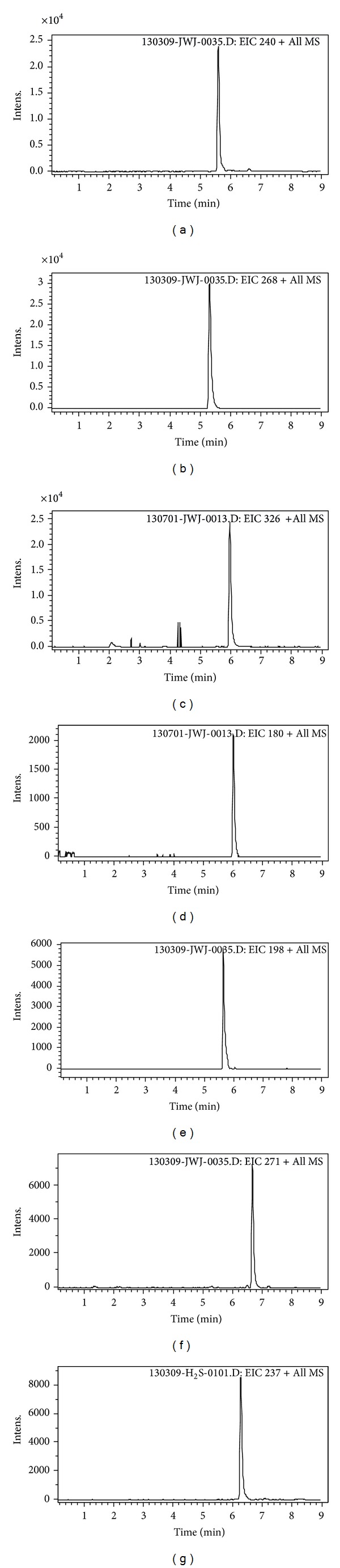
LC-MS chromatograms: blank plasma spiked with bupropion (1), metoprolol (2), midazolam (3), phenacetin (4), omeprazole (5), tolbutamide (6), and carbamazepine (IS) (7).

Figure 1 showed the typical chromatograms of a blank plasma sample spiked with probe drugs and IS detected by LC-MS. No interfering endogenous substances were observed at the retention time of the analytes and IS. This demonstrates that the chromatographic separation method has a good peak shape and resolution.

Calibration curves for six probe drugs were generated by linear regression of peak area ratios against concentrations, respectively. The calibration plots of the probe drugs in the range of 10–2000 ng/mL were listed in Table 1. Each probe drug peak area ratio with concentration has a good linear relationship with the range of concentration. The LLOQ for each probe drug in plasma was 10 ng/mL.

Table 2 showed the results of intraday precision, interday precision, accuracy, extraction recovery, and matrix effect. The relative standard deviation (RSD%) of the six probe drugs in low, medium, and high, three, concentrations was less than 15%. The intraday and interday accuracy ranged from 88.5% to 111.2%. The extraction recoveries were ranged from 83.4% to 94.2%. The results of matrix effect and the percent nominal concentration were more than 85.5% or less than 111.8%. The results indicate that ion suppression or enhancement from the plasma matrix was negligible for this analytical method.

### 3.2. Pharmacokinetic Study

The main pharmacokinetic parameters after administration of bupropion, metoprolol, midazolam, phenacetin, omeprazole, and tolbutamide from noncompartment model analysis were summarized in Table 3. In the experiment for acute H_2_S poisoning and control group, there was a statistically significant difference in the AUC and *C*_max⁡_ for bupropion, metoprolol, phenacetin, and tolbutamide (*P* < 0.05), while there was no statistical difference for midazolam and omeprazole (*P* > 0.05).

As can be seen from Table 3, comparing acute H_2_S poisoning group with the control group, the pharmacokinetic parameters of bupropion have changed; *t*_1/2_ from the 0.9 h increased to 1.0 h, but there was no statistical significance (*P* > 0.05); AUC_(0–*t*)_ from the 417.3 increased to 650.5 ng/mL∗h, with significant difference (*P* < 0.05); CL from 27.3 reduced to 16.3 L/h/kg, with statistical significance (*P* < 0.05); *C*_max⁡_ varied from 276.6 to 411.3 ng/mL, with statistical significance (*P* < 0.05). Comparing acute H_2_S poisoning group with the control group, the pharmacokinetic parameters of metoprolol have changed; AUC_(0–*t*)_ from the 1346.2 increased to 2182.9 ng/mL∗h, with significant difference (*P* < 0.01); CL from 9.5 reduced to 4.9 L/h/kg, with no statistical significance (*P* < 0.05); *C*_max⁡_ varied from 810.8 to 1235.7 ng/mL, with statistical significance (*P* < 0.01). Comparing acute H_2_S poisoning group with the control group, the pharmacokinetic parameters of midazolam have almost not changed; *C*_max⁡_ varied from 1343.6 to 1353.0 ng/mL, and there no statistical significance (*P* > 0.05). Comparing acute H_2_S poisoning group with the control group, the pharmacokinetic parameters of phenacetin have changed; *t*_1/2_ from the 0.6 h reduced to 0.4 h, and there was statistical significance (*P* < 0.05); AUC_(0–*t*)_ from the 1239.9 increased to 1993.4 ng/mL∗h with significant difference (*P* < 0.05); CL from 11.1 reduced to 5.4 L/h/kg, but there was no significant difference (*P* > 0.05); *C*_max⁡_ varied from 1329.2 to 1847.7 ng/mL, and there was significant difference (*P* < 0.01). Comparing acute H_2_S poisoning group with the control group, the pharmacokinetic parameters of omeprazole have almost not changed; *t*_1/2_ from the 1.1 h reduced to 0.8 h, and there no statistical significance (*P* > 0.05). Comparing acute H_2_S poisoning group with the control group, the pharmacokinetic parameters of tolbutamide have changed; *t*_1/2_ from the 3.9 h increased to 4.2 h, but there was no statistical significance (*P* > 0.05); AUC_(0–*t*)_ from the 11280.4 increased to 16139.1 ng/mL∗h, with significant difference (*P* < 0.05); CL from 0.10 reduced to 0.06 L/h/kg, and there was statistical significance (*P* < 0.05); *C*_max⁡_ varied from 1689.3 to 2216.5 ng/mL, and there was statistical significance (*P* < 0.05).

**Figure 2:**
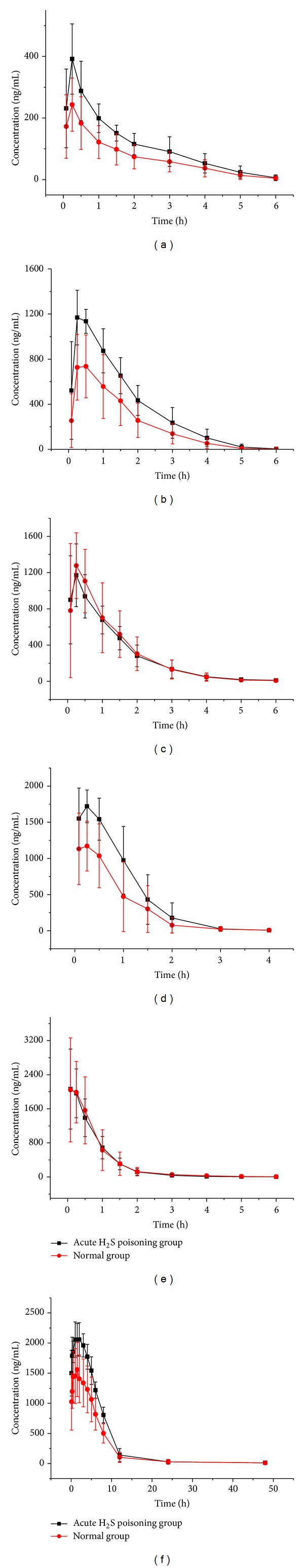
The pharmacokinetics profiles of bupropion (a), metoprolol (b), midazolam (c), phenacetin (d), omeprazole (e), and tolbutamide (f) after oral administration in acute hydrogen sulfide poisoning group and control group rats (*n* = 8).

The representative bupropion, metoprolol, midazolam, phenacetin, omeprazole, and tolbutamide concentration versus time profiles of twelve rats were presented in Figure 2. As could be seen from Figure 2, the AUC and *C*_max⁡_ of bupropion, metoprolol, phenacetin, and tolbutamide in acute H_2_S poisoning group are higher than the control group; this result is consistent with the Table 3. The concentration-time curve diagram of midazolam and omeprazole in acute H_2_S poisoning group almost coincided with control group.

## 4. Discussion

Compared to the control group, in acute H_2_S poisoning group, the AUC bupropion goes higher (*P* < 0.05), CL decreases (*P* < 0.05), and *C*_max⁡_ becomes higher (*P* < 0.05); this indicates that acute H_2_S poisoning will inhibit the activity of CYP2B6 enzyme. Similar results were found for tolbutamide; the AUC_(0–*t*)_ increased (*P* < 0.05); CL reduced (*P* < 0.05); *C*_max⁡_ became higher (*P* < 0.05); this indicates that acute H_2_S poisoning will inhibit the activity of CYP2C9 enzyme.

Compared to the control group, in the acute H_2_S poisoning group, the AUC_(0–*t*)_ of metoprolol increased (*P* < 0.01); *C*_max⁡_ became higher (*P* < 0.01); this indicates that acute H_2_S poisoning will inhibit the activity of CYP2D6 enzyme. Similar results were found for phenacetin; AUC_(0–*t*)_ increased (*P* < 0.05); *C*_max⁡_ became higher (*P* < 0.01); this indicates that acute H_2_S poisoning will inhibit the activity of CYP1A2 enzyme.

Compared to the control group, in the acute H_2_S poisoning group, the pharmacokinetic parameters of midazolam have almost not changed; this indicates that the acute H_2_S poisoning will not induce or inhibit the activity of CYP3A4 enzyme. Similar results were found for omeprazole; the pharmacokinetic parameters of omeprazole have almost not changed between control group and acute H_2_S poisoning group; this shows that the acute H_2_S poisoning will not induce or inhibit the activity of CYP2C19 enzyme.

The results demonstrated that acute H_2_S poisoning could inhibit the activity of CYP2B6, CYP2D6, CYP1A2, and CYP2C9 of rats. The results may make sense for the clinical oral use of drugs for the people after acute H_2_S poisoning. Drugs are metabolized by CYP2B6, CYP2D6, CYP1A2, and CYP2C9 enzymes after acute H2S poisoning; we should pay close attention to changes in the plasma concentration to avoid drug interactions that may occur.

## 5. Conclusion

The concentrations of probe drugs in rat plasma were successfully measured by LC-MS. In the experiment for acute H_2_S poisoning and control group, there was a statistically significant increase in the AUC and *C*_max⁡_ for bupropion, metoprolol, phenacetin, and tolbutamide, while there was no statistical pharmacokinetics difference for midazolam and omeprazole. Acute H_2_S poisoning could inhibit the activity of CYP2B6, CYP2D6, CYP1A2, and CYP2C9 of rats. The results may make sense for the clinical oral use of drugs for the people after acute H_2_S poisoning.

## Figures and Tables

**Table 1:** Regression equation and correlation coefficient for six probe drugs.

Probe drugs	Liner range (ng/mL)	Regression equation	Correlation coefficient
Bupropion	10–2000	*y* = 0.002629*x* + 0.055076	0.997233
Metoprolol	10–2000	*y* = 0.003557*x* + 0.110593	0.997110
Midazolam	10–2000	*y* = 0.003584*x* + 0.146928	0.996311
Phenacetin	10–2000	*y* = 0.000656*x* − 0.001949	0.998495
Omeprazole	10–2000	*y* = 0.000602*x* + 0.032108	0.996069
Tolbutamide	10–2000	*y* = 0.000576*x* + 0.012733	0.995463

y: peak area ratio of probe drugs versus IS; *x*: concentration of probe drugs.

**Table 2:** Precision, accuracy, recovery, and matrix effect of six probe drugs in rat plasma (mean ± SD, *n* = 6).

Compound	Concentration (ng/mL)	Precision RSD (%)		Accuracy (%)		Recovery	Matrix effect
		Intraday	Interday	Intraday	Interday		
Bupropion	20	6.7	9.1	102.6	102.8	83.4 ± 5.1	111.8 ± 4.7
	200	3.2	7.6	101.7	94.6	87.4 ± 4.9	101.2 ± 4.8
	1600	2.6	9.3	97.6	106.8	91.5 ± 4.7	96.4 ± 3.3

Metoprolol	20	13.3	13.4	104.5	94.8	84.9 ± 6.9	109.1 ± 4.7
	200	5.4	9.3	93.8	95.9	87.5 ± 5.8	106.3 ± 3.9
	1600	6.4	6.9	93.1	103.6	90.1 ± 4.4	97.2 ± 3.8

Midazolam	20	7	9.4	103.4	109.9	90.3 ± 7.5	104.3 ± 4.9
	200	6.6	5.9	92.3	88.7	92.1 ± 6.8	102.4 ± 5.5
	1600	2.6	6.5	96.2	106.3	94.2 ± 5.6	99.1 ± 4.8

Phenacetin	20	6.3	8.6	109.5	111.2	88.6 ± 5.7	88.2 ± 6.2
	200	11.6	10.2	105.7	96.3	86.6 ± 7.3	90.5 ± 6.8
	1600	3.4	7.6	97.8	105.2	92.6 ± 4.3	87.4 ± 4.2

Omeprazole	20	12.8	14.8	88.5	98.7	93.1 ± 9.2	86.7 ± 6.4
	200	5	7.7	91.7	104.5	88.7 ± 5.6	89.5 ± 4.7
	1600	4.9	8.6	94.5	105.1	90.2 ± 7.1	85.5 ± 6.7

Tolbutamide	20	11.4	13.5	91.8	103.4	89.1 ± 7.8	90.1 ± 4.8
	200	3.5	6	100.8	90.7	93.8 ± 5.6	87.5 ± 7.1
	1600	6.1	7.2	105.3	93.6	94.1 ± 4.7	85.3 ± 6.5

**Table 3:** Pharmacokinetic parameters of six probe drugs after oral administration in acute hydrogen sulfide poisoning group and control group rats (mean ± SD, *n* = 8).

Probe drugs	Parameters	AUC_(0–*t*)_	AUC_(0–*∞*)_	MRT_(0–*t*)_	MRT_(0–*∞*)_	*t*1/2*z*	*T*_max⁡_	*CL* *z*/*F*	*Vz*/*F*	*C*_max⁡_
	Unit	ng/mL∗h	ng/mL∗h	h	h	h	h	L/h/kg	L/kg	ng/mL
Bupropion	H_2_S	650.5 ± 208.4*	671.1 ± 225.9*	1.6 ± 0.2	1.8 ± 0.3	1.0 ± 0.3	0.2 ± 0.1	16.3 ± 5.1	21.6 ± 6.4	411.3 ± 113.3*
	Control	417.3 ± 192.6	427.3 ± 197.8	1.6 ± 0.2	1.7 ± 0.2	0.9 ± 0.4	0.2 ± 0.1	27.3 ± 10.9	33.9 ± 16.9	276.6 ± 72.3

Metoprolol	H_2_S	2182.9 ± 508.6**	2188.4 ± 510.5**	1.4 ± 0.2	1.4 ± 0.2	0.6 ± 0.1	0.3 ± 0.1	4.9 ± 1.5	3.9 ± 1.6	1235.7 ± 160.8**
	Control	1346.2 ± 593.6	1348.0 ± 593.2	1.3 ± 0.2	1.3 ± 0.2	0.5 ± 0.2	0.7 ± 0.5	9.5 ± 6.2	8.5 ± 8.9	810.8 ± 279.8

Midazolam	H_2_S	1703.7 ± 504.5	1715.4 ± 503.9	1.2 ± 0.1	1.3 ± 0.2	0.9 ± 0.5	0.2 ± 0.1	6.3 ± 2.0	9.0 ± 9.3	1253.0 ± 318.2
	Control	1811.1 ± 834.3	1819.9 ± 832.1	1.2 ± 0.1	1.2 ± 0.1	0.8 ± 0.3	0.2 ± 0.1	6.5 ± 2.8	8.5 ± 6.3	1343.6 ± 408.8

Phenacetin	H_2_S	1993.4 ± 667.3*	1998.1 ± 668.8*	0.7 ± 0.1	0.7 ± 0.2	0.4 ± 0.1*	0.3 ± 0.2	5.4 ± 1.6	2.9 ± 0.8	1847.7 ± 219.4**
	Control	1239.9 ± 675.8	1243.9 ± 677.7	0.7 ± 0.2	0.7 ± 0.2	0.6 ± 0.2	0.3 ± 0.2	11.1 ± 8.1	10.6 ± 12.1	1329.2 ± 397.6

Omeprazole	H_2_S	1826.6 ± 592.1	1830.1 ± 591.2	0.7 ± 0.1	0.7 ± 0.1	0.8 ± 0.3	0.1 ± 0.1	6.3 ± 3.0	7.7 ± 5.6	2248.6 ± 691.7
	Control	1919.3 ± 1013.1	1929.4 ± 1010.3	0.8 ± 0.1	0.8 ± 0.1	1.1 ± 0.3	0.2 ± 0.1	6.3 ± 2.6	10.2 ± 5.5	2357.7 ± 882.9

Tolbutamide	H_2_S	16139.1 ± 1941.3*	16240.0 ± 2149.2*	5.4 ± 1.6	5.7 ± 2.3	4.2 ± 2.4	1.9 ± 0.8	0.06 ± 0.01*	0.4 ± 0.2*	2216.5 ± 229.3*
	Control	11280.4 ± 3894.9	11290.6 ± 3906.9	5.6 ± 1.3	5.6 ± 1.3	3.9 ± 1.1	1.3 ± 0.8	0.10 ± 0.04	0.5 ± 0.2	1689.3 ± 503.0

Compared with the control group, *P < 0.05 and **P < 0.01.
